# Prior Cancer among Men Diagnosed with Breast Cancer is Associated with Worse Overall Survival but Equivalent Breast Cancer-Specific Survival

**DOI:** 10.7150/jca.119554

**Published:** 2026-03-25

**Authors:** Aniruddha Rathod, Caitlin C. Murphy, Kathryn L. Shahan, Sandi L. Pruitt

**Affiliations:** 1 Department of Epidemiology, Fay W. Boozman College of Public Health, University of Arkansas for Medical Sciences, Little Rock, AR, USA.; 2 Winthrop P. Rockefeller Cancer Institute, University of Arkansas for Medical Sciences, Little Rock, AR, USA.; 3 Peter O'Donnell Jr. School of Public Health, University of Texas Southwestern Medical Center, Dallas, TX, USA.; 4 Department of Pediatrics, University of Chicago, Chicago, IL, USA.; 5 UChicago Medicine Comprehensive Cancer Center, University of Chicago, Chicago, IL, USA.; 6 Harold C. Simmons Comprehensive Cancer Center, University of Texas Southwestern Medical Center, Dallas, TX, USA.

**Keywords:** male breast cancer, multiple primary malignancies, prior cancer, SEER, clinical trials, survival

## Abstract

**Background:**

Men newly diagnosed with breast cancer (BC) who have survived prior cancer are often excluded from clinical trials, despite limited evidence on how prior cancer impacts BC outcomes. Understanding survival of those with prior cancer is crucial for trial sponsors and investigators to make informed eligibility decisions. Using Surveillance, Epidemiology, and End Results (SEER) data, we investigated the impact of prior cancer on survival in men diagnosed with BC between 2011 and 2015.

**Methods:**

We evaluated the prevalence of prior cancer of a different type in adult men with BC and analyzed its effects on overall and BC-specific survival using Cox models and the Fine and Gray method to account for competing risks of death. Models adjusted for covariates including patient, tumor, and treatment factors.

**Results:**

Among 1,923 men with BC, 241 (12.5%) had a prior cancer of different type. There were 904 deaths from any cause including 365 from BC. Median overall survival was 10.1 years. Adjusted models demonstrated worse overall survival for men with prior cancer (HR=1.3, 95% CI: 1.1, 1.6) but no statistically significant difference in BC-specific survival (sdHR=0.9, 95% CI: 0.7, 1.4) between the groups.

**Conclusions:**

Despite worse overall survival, men with prior cancer did not have worse BC-specific survival, suggesting their inclusion in trials is unlikely to introduce bias in BC-specific survival.

**Implications for Cancer Survivors:**

Including men with prior cancer in trials may enhance trial accrual, inclusivity, and generalizability, critical aspects of research studies for this rare cancer in which accrual is already a challenge.

## Introduction

There were an estimated 18.1 million cancer survivors in the United States as of January 1, 2022 1, a number projected to exceed 22 million by 2030 due to advances in cancer treatment and an aging population 2. As the survivor population grows, the incidence of second primary cancers is also increasing, creating a subgroup of survivors with unique clinical needs and need for specialized survivorship services to address their complex care considerations. Cancer survivors with a new primary malignancy are frequently excluded from clinical trials, limiting the applicability and generalizability of study findings to this population. For instance, a review of breast cancer (BC) clinical trial protocols found that the majority (66%) excluded patients with a prior cancer, and almost half (47%) explicitly required a five-year cancer-free interval before enrollment 3. Similarly, an analysis of 87 NCI-sponsored BC clinical trials between 1991-2016 reported that 77% excluded survivors with a prior cancer, most commonly (79%) when the cancer was diagnosed within the preceding five years 4. These restrictive eligibility criteria may be especially detrimental in trials for rare cancers, such as male BC, where accrual is already challenging, further limiting evidence generation and applicability to real-world populations.

Male BC is a rare cancer accounting for less than 1% of all BC diagnoses 5. About 2800 new cases of invasive BC will be diagnosed among men in the United States in 2023, 6 with a 5-year relative survival of 82%. Our previous study demonstrated that nearly one quarter of men diagnosed with BC between 2011-2015 had survived a prior cancer 7, highlighting the need for inclusive research practices. Including these cancer survivors in clinical trials may increase the generalizability of findings and ultimately improve evidence-based treatments. Additionally, due to the rarity of male BC, accruing participants for clinical trials is a major challenge, and excluding cancer survivors without sufficient rationale only adds to these accrual challenges. In a comprehensive review of 426 BC clinical trials active between January 1, 2000, and April 30, 2017, 65% explicitly excluded male patients, and overall, only 0.42% of participants were men. Notably, none of the 70 trials investigating neoadjuvant therapy enrolled male patients, and only five studies designed to include men were completed 8. There is limited evidence about the prognostic impact of prior cancer on outcomes, including survival, for men diagnosed with BC. Understanding whether men with prior cancer have better or worse overall survival and BC-specific survival compared to those without prior cancer is important for designing inclusive clinical trials and for informing clinical care practices. In this study, we examined the impact of prior cancer on overall survival and BC-specific survival in a population-based sample of men diagnosed with BC, with the ultimate goal of contributing data to inform evidence-based trial eligibility criteria.

## Methods

*Data.* We used population-based data from National Cancer Institute's Surveillance, Epidemiology, and End Results (SEER) program of cancer registries 9. We included adult men (aged ≥ 18 years) diagnosed with an index BC between 2011-2015 from the SEER 17 database, ensuring a minimum of 5 years of follow-up to assess long-term survival outcomes. For men with more than one BC diagnosed in 2011-2015 (n = 46), the first diagnosis in the study period was selected as the index BC.

*Measures.* Prior cancer was defined as having any primary cancer prior to diagnosis of the index BC, and we used the SEER variables *sequence number,* i.e., order of all primary tumors, and diagnosis year, to identify prior cancers. We included the following two groups of men with BC: 1) Men without prior cancer, i.e., diagnosed with only the index BC, and 2) Men with prior cancer, i.e., diagnosed with one prior cancer of a different cancer type before the index BC. We excluded 294 men with prior cancer (29 with prior BC, 169 with prior cancer of an unknown type, and 96 with more than one prior cancer of a different type) to ensure accurate measurement of cancer-specific survival from the index BC.

*Analysis.* We estimated prevalence of prior cancer of a different type and described characteristics of men with and without prior cancer, including age at index BC, race and ethnicity, median household income at the county level, marital status, rural/urban residence, BC stage (AJCC 7^th^ edition), tumor size (in centimeters), hormone receptor type, histology, receipt of chemotherapy and radiation (yes/no/unknown), type of surgery (mastectomy or breast conserving surgery) if any, vital status, BC-specific death, type of prior cancer, and time elapsed between prior cancer and index BC. We compared these characteristics using t-test and chi-square or Fisher's exact tests.

Our outcomes were overall and BC-specific survival. Overall survival was measured in months from the index BC diagnosis to death from any cause or last follow-up date (December 31, 2022). BC-specific survival was measured from the index BC diagnosis to death specifically attributed to BC, with non-BC deaths treated as competing risks. Men with missing survival time were excluded (n=12). Cause of death in SEER is derived from death certificates, and the SEER Cause of Death Recode variable is considered accurate for measuring cause-specific death; for example, BC-specific death has a high accuracy of 98.1% 10 and, more recently, methods have ensured that cause of death coding is accurate for individuals with multiple tumors.^11^

We plotted cumulative incidence curves to compare the cumulative incidence of all-cause and BC-specific death between men with and without prior cancer. Additionally, we also plotted cumulative incidence of death from any cancer (except BC) among men with prior cancer. We estimated cumulative incidence of all-cause death (death due to any cause) using the Kaplan-Meier method. We modeled the hazard of the all-cause mortality using unadjusted and adjusted Cox proportional hazard regression. We included age at BC diagnosis, BC stage, and tumor size as covariates in the adjusted model based on association of prior cancer with the covariates (see Table 1) and previous research on prior cancer 12, using the same categories as presented in Table 1. The hazard ratios (HR) and 95% confidence intervals from crude and adjusted models quantified the association between prior cancer and overall survival. We evaluated the proportional hazard assumption by including time dependent variables in the model.

Further, we estimated cumulative incidence of BC-specific death using the Fine and Gray model 13 to account for the competing risks of death due to other causes. For BC-specific death, we modeled the hazard of cumulative incidence function using Fine and Gray proportional subdistribution hazard regressions. The subdistribution hazard ratios (sdHR) and 95% confidence intervals from crude and adjusted models quantified the association between prior cancer and BC-specific survival.

We conducted a subgroup analysis focusing on BC patients with a history of prostate cancer, the most common type of prior cancer. We also conducted a sensitivity analysis to study the association of time elapsed between prior cancer and index BC with survival, as it might play a role in influencing patient outcomes. Finally, we also conducted subgroup analysis to evaluate the association of prior cancer stage on survival.

## Results

**Figure 1:**
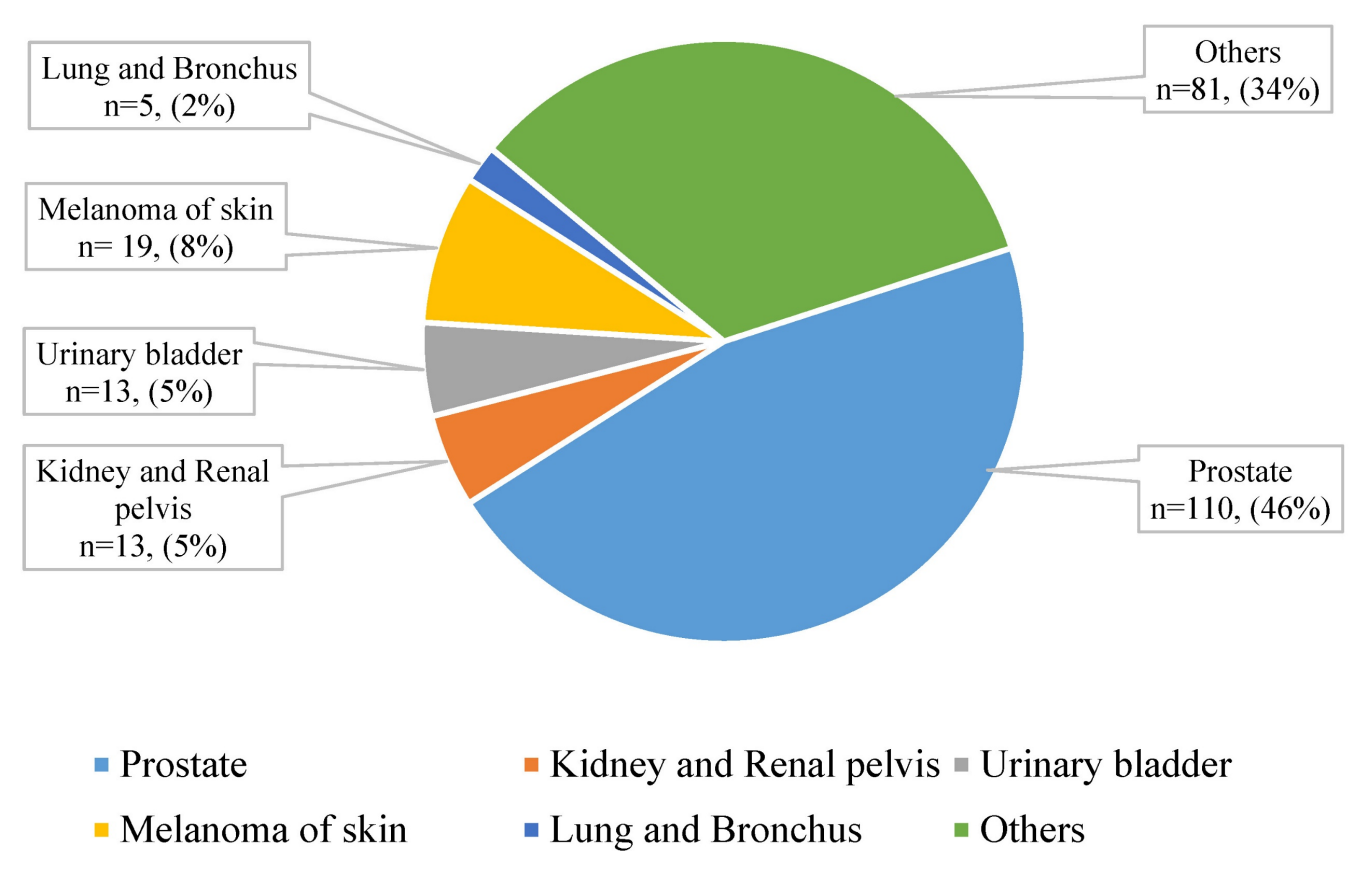
Prior cancer type among men diagnosed with breast cancer and with one prior cancer (n=241).

We identified 1923 men diagnosed with BC during 2011-2015, of whom 241 (12.5%) had prior cancer of a different type. The differences in characteristics of men with and without prior cancer are summarized in Table 1. Men with prior cancer were older, had smaller (≤ 2 cm) breast tumors, and were less likely to receive chemotherapy and radiation compared to men without any prior cancer. Figure 1 represents the most common types of prior cancer among men diagnosed with BC (n=241), including prostate cancer, kidney and renal pelvis, urinary bladder, and others. We observed 904 deaths from any cause including 365 deaths due to BC.

**Figure 2:**
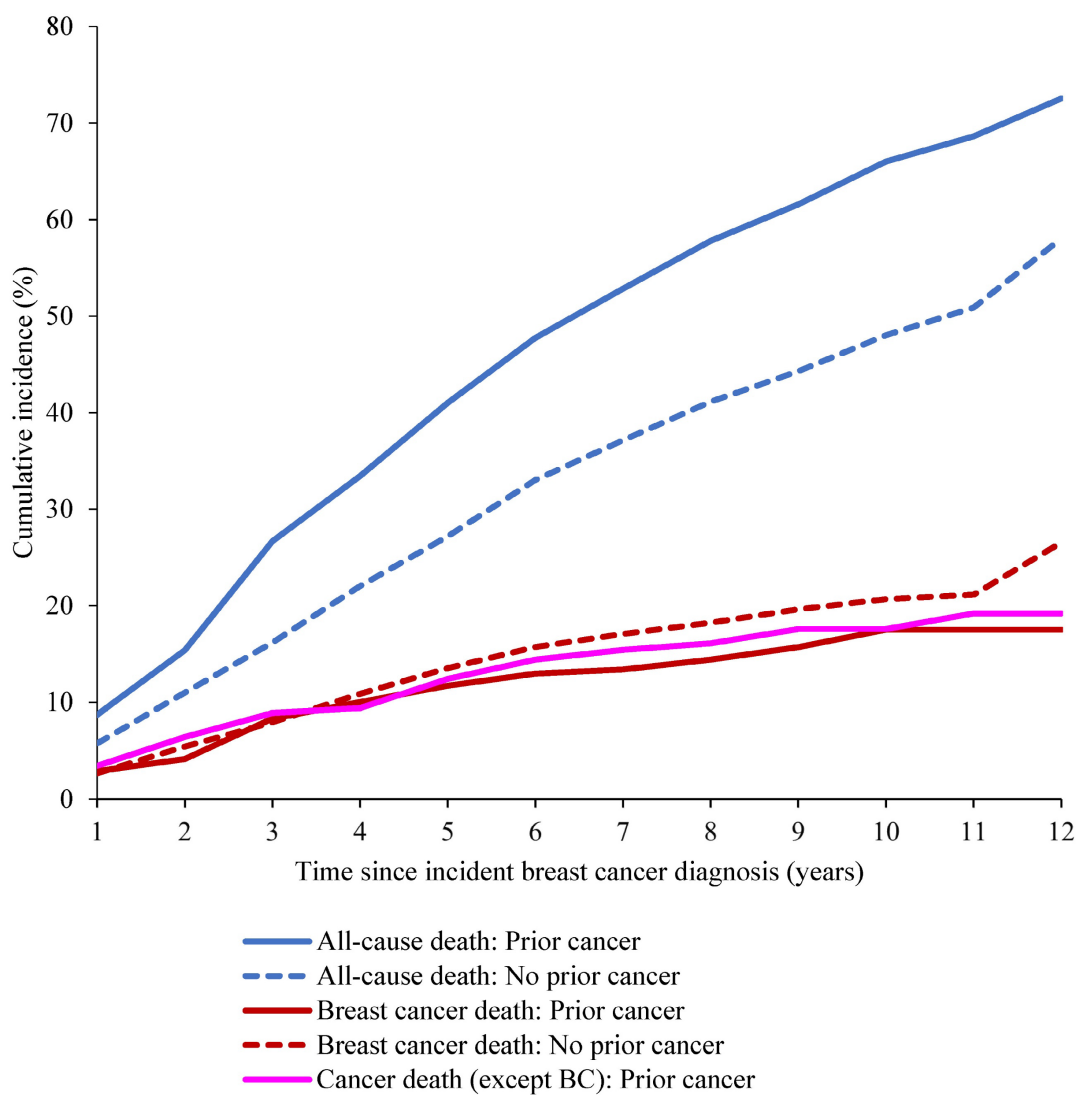
Cumulative incidence of breast cancer (cause-specific) and all-cause death.

Figure 2 and Table 2 show cumulative incidence of all-cause death and BC-specific death. The cumulative incidence of all-cause death was 41% and 27.2% at five years after BC diagnosis for men with and without prior cancer, respectively. The cumulative incidence of death from any cancer (except BC) among men with prior cancer was similar to the cumulative incidence of BC-specific death (Figure 2).

In the adjusted Cox models, men with prior cancer had worse overall survival (HR = 1.31, 95% CI: 1.09, 1.57) compared to those without prior cancer (Table 3). After accounting for competing risks, men with prior cancer had equivalent risk of BC-specific death compared to men without prior cancer (sdHR = 0.94, 95% CI: 0.66, 1.35).

Because prostate cancer was the most common prior cancer, we conducted a subgroup analysis focusing on BC patients with a history of prostate cancer (Table 3, n = 1792). We did not observe statistical significance for adjusted Cox models for overall survival (HR = 1.01, 95% CI: 0.77, 1.32) nor BC-specific survival (sdHR=0.57, 95% CI: 0.31, 1.05) for those with prior prostate cancer compared to those without prior cancer. Additionally, we conducted another subgroup analyses to study the association of time elapsed between prior cancer and index BC with survival. Men whose prior cancer occurred within 5 years of their index BC diagnosis experienced better BC-specific survival (sdHR = 0.44, 95% CI: 0.20, 0.94) compared to > 5 years. We did not observe statistical significance for overall survival (HR = 1.08, 95% CI: 0.77, 1.52) for ≤ 5 years compared to > 5 years. Lastly, we conducted a subgroup analysis to study the association of prior cancer stage at diagnosis with survival. Men whose prior cancer was diagnosed at stage IV (n = 25) had worse overall survival (HR = 2.76, 95% CI: 1.62, 4.69) compared to prior cancer diagnosed at stage 0/I (Supplement table). We did not observe effects of prior cancer stage on BC-specific survival.

## Discussion

In this study, using US population-based data, we demonstrated that 12.5% of men newly diagnosed with BC had survived a prior cancer of a different type. This finding aligns with studies demonstrating a substantial, and increasing, prevalence of prior cancer 14 and underscores the importance of understanding how prior cancer impacts subsequent outcomes. Little is known about the impact of prior cancer on survival outcomes, and we addressed this gap by examining the impact of prior cancer on survival of men with BC. We showed that men with prior cancer had worse overall survival compared to those without prior cancer. Men with BC and a prior cancer present with additional factors influencing their survival, such as older age, the long-term effects of prior cancer treatment such as cardiotoxicity, nephrotoxicity and neurotoxicity 15, other unknown comorbidities, or progression of the prior cancer. These factors should be considered when designing and conducting clinical trials and treatment strategies, as prior cancer could affect the interpretation of survival outcomes. From a clinical care perspective, it is important to recognize that prior cancer history may necessitate tailored follow-up and management strategies for survivors facing a new cancer diagnosis such as more frequent surveillance, multidisciplinary care, psychosocial and supportive care, and additional support when making treatment decisions.

In contrast, we found BC-specific survival was equivalent between men with and without prior cancer. This observation is clinically relevant, as it indicates that although overall survival may be lower, the prognosis related specifically to BC is not adversely affected. In subgroup analyses, men with prior cancer, whose prior malignancy occurred within 5 years of their index BC diagnosis, experienced better BC-specific survival compared to those with a time interval of > 5 years. However, this finding must be interpreted with caution, it may be subject to survivorship bias as only men who survived prior cancer long enough to develop a new BC were included. Furthermore, men whose prior cancer was diagnosed at stage IV had substantially worse overall survival than those diagnosed at stage 0/I; however, no such association was observed for BC-specific survival. Taken together, these findings suggest that prior cancer characteristics do not appear to compromise BC-specific outcomes.

For clinical sponsors and investigators, these findings support the rationale for including men with prior cancer in clinical trials, particularly given that most trials evaluate BC-specific endpoints. Most clinical trials of men with BC are designed to evaluate treatment efficacy, thus BC-specific survival is an important outcome. This should give trial sponsors and investigators pause before deciding to exclude men with prior cancer from clinical trials. While it is understandable that factors such as older age and comorbidities are common among those with prior cancer, these are often cited as reasons for exclusion, and such practices can inadvertently limit the generalizability of trial findings. Excluding this subgroup may result in trial outcomes that do not fully represent the broader population of men with BC, thus undermining the applicability of results to those who will ultimately be treated. Additionally, the exclusion of men with prior cancer may obscure important variations in patient outcomes, given our findings regarding differences in characteristics of those with vs. without prior cancer. Consequently, these exclusions may hinder the ability to draw conclusions about the effectiveness of interventions across different patient profiles, ultimately reducing the robustness and applicability of clinical trial evidence. Ideally, clinical trials should reflect real-world patient populations and allow for evaluation of treatment efficacy in diverse populations.

Increasing trial participation and accrual is important for all cancer types, and especially so for men with BC. A study examining the inclusion and representation of men in BC clinical trials between 2000 and 2017 found that men represented only 0.42% of all participants, and no published studies successfully completed participant recruitment 8. Because male BC is rare and the limited number of trials specifically designed for men means that treatment recommendations are often extrapolated from studies conducted in women 16. This reliance is challenging because important biological differences exist between male and female breast tissue 17. Although BC in both sexes is predominantly ductal, breast development diverges markedly after puberty. In women, ducts elongate and branch, and lobules form under hormonal influence, whereas male breast tissue remains rudimentary, containing ducts but very few lobules and consisting largely of adipose tissue. Moreover, breast epithelial cells in women are hormonally active and responsive to estrogen, while those in men are relatively inactive and not exposed to sustained high estrogen levels 18. Male BC is more likely to express hormone receptors than female BC 19. In addition to these anatomical and hormonal distinctions, men with BC have worse overall survival than women 20. These gaps highlight the importance of increasing clinical trial accrual and discovery of effective treatments for men with BC.

Amongst those who had prior cancer, the cumulative incidence of death due to cancer (except BC) was comparable to BC-specific death. This suggests that the notable difference between cumulative incidence of all-cause mortality and BC-specific mortality is partially, but not entirely, driven by non-BC-deaths including deaths from prior cancer. We also observed worse overall survival among men with advanced prior cancer stage. Together, these findings warrant further investigation into the mechanisms driving mortality in patients with prior cancer.

The American Society of Clinical Oncology recommends including patients with prior cancer in clinical trials when a) the risk of prior cancer interfering with either safety or efficacy end points is very low, and b) if prior cancer treatment was completed at least 2 years before registration and patient has no evidence of disease 21. After considering our findings regarding BC-specific and overall survival differences for men with and without prior cancer, trial sponsors and investigators should consider including these men who can benefit from novel therapies and contribute valuable data to clinical studies, particularly given difficulty of accruing adequate numbers of men with BC into clinical trials.

Our study includes several strengths. We utilized large US population-based cancer registry data with available patient, tumor, and treatment characteristics. We accounted for competing risks in our analysis, such as death from a prior cancer or other causes, which provides more clinically relevant survival estimates. Our population-based study is unique given our focus on prognostic impact of prior cancer on survival of men with BC, and to our knowledge is the first study on the topic in this rare and understudied cancer in which clinical trial accrual is already a challenge. One of the limitations of our study is possible misclassification of cause of death which may affect our competing risk analysis. To lessen this potential misclassification, we restricted our analysis to include men with only one prior cancer, and we note that this limitation does not impact our results about overall survival in which we analyzed deaths from any cause. Given the rarity of this cancer and the wide 95% CI for BC-specific survival, we acknowledge the limited statistical power to detect differences in BC-specific survival between the two groups. In addition, there are limitations related to treatment data in the SEER dataset that precluded our ability to examine relationships among treatment for prior cancer, treatment for MBC, and survival. First, SEER combines “no” and “unknown” categories for chemotherapy and radiation therapy, and “unknown” does not necessarily indicate the absence of treatment. Second, there is no detail on type, dose, or duration of therapy, and these factors may be especially relevant to survival. In addition, we were unable to account for germline cancer-predisposition variants (e.g., *BRCA1/2, PALB2, CHEK2*) or biological changes resulting from treatments for patients' prior cancers, such as homologous recombination deficiency (HRD) or other DNA-repair defects, due to SEER's lack of genetic and biomarker data. These factors may influence both the development of BC and its survival; HRD status also functions as an eligibility criterion and predictive biomarker in clinical trials. Future studies related to clinical trial eligibility criteria and the epidemiology and outcomes of men diagnosed with BC as well as other cancer types should incorporate genetic and HRD profiling.

In conclusion, given our finding of worse overall survival but equivalent BC-specific survival among men with vs. without prior cancer, men with prior cancer should be considered for participation in clinical trials and provided comprehensive clinical care tailored to their unique survivorship needs.

## Figures and Tables

**Table 1:** Characteristics among men with breast cancer, comparing men with a prior cancer of a different type to those without prior cancer.

Characteristics	With prior cancer (n = 241) n (%)	No prior cancer (n = 1682) n (%)	p-Value
Age at breast cancer diagnosis (in years)			< 0.01^a^
Mean (SD)	72.7 (9.4)	65.4 (12.1)	
Median (IQR)	73 (14)	66 (16)	
Race and ethnicity			0.22
Non-Hispanic White	188 (78)	1211 (72)	
Non-Hispanic Black	28 (11.6)	223 (13.3)	
Hispanic White	12 (5)	138 (8.2)	
Other	13 (5.4)	101 (6)	
Missing	0	9	
Median household income (in thousands) at county level (in USD)			0.36
< 50	12 (5)	121 (7.2)	
50 to 70	50 (20.8)	372 (22.1)	
> 70	179 (74.3)	1188 (70.7)	
Missing		1	
Marital Status			0.05
Single	22 (9.1)	257 (15.3)	
Married/Unmarried or domestic partner	161 (66.8)	1094 (65)	
Separate/divorced/widowed	41 (17)	224 (13.3)	
Missing	17	107	
Rural/urban residence at county level			0.73
Rural	25 (10.4)	187 (11.1)	
Urban	216 (89.6)	1494 (88.9)	
Missing		1	
Breast cancer stage (AJCC 7^th^ edition)			0.18
0-I	91 (37.8)	512 (30.4)	
IIA	63 (26.1)	457 (27.2)	
IIB	32 (13.3)	247 (14.7)	
III	27 (11.2)	275 (16.4)	
IV	19 (7.9)	131 (7.8)	
Missing	9	60	
Tumor size (in cm)			0.01
No mass or tumor found^#^	3 (1.3)	4 (0.3)	
≤ 1	22 (9.6)	149 (9.4)	
> 1 to ≤ 2	92 (40.2)	548 (34.5)	
> 2 to ≤ 5	106 (46.3)	784 (49.4)	
> 5	6 (2.6)	102 (6.4)	
Missing	12	95	
Hormone receptor type			0.5^b^
Luminal A	195 (88.2)	1309 (86.5)	
Luminal B	21 (9.5)	165 (10.9)	
HER-2 enriched	3 (1.4)	11 (0.7)	
Triple negative	2 (0.9)	28 (1.9)	
Missing	20	169	
Histology			0.23
Infiltrating duct	209 (86.7)	1500 (89.2)	
Other Adenocarcinoma	24 (10)	117 (7)	
Other	8 (3.3)	65 (3.9)	
Surgery			0.13^b^
Mastectomy	187 (77.6)	1351 (80.3)	
Breast conserving surgery	23 (9.5)	161 (9.6)	
None	29 (12)	168 (10)	
Missing	2	2	
Chemotherapy			< 0.01
Yes	63 (26.1)	686 (40.8)	
No/Unknown	178 (73.9)	996 (59.2)	
Radiation			< 0.01
Yes	48 (19.9)	493 (29.3)	
No/Unknown/Refused	193 (80.1)	1189 (70.7)	
Vital Status			< 0.01
Alive	91 (37.8)	928 (55.2)	
Dead	150 (62.2)	754 (44.8)	
Breast Cancer specific death			0.19
Yes	38 (15.8)	327 (19.4)	
No	203 (84.2)	1355 (80.6)	
Time elapsed between prior cancer and index breast cancer			
≤ 5 years	136 (56.4)	-	
> 5 years	105 (43.6)	-	
Top 5 prior cancer sites			
Prostate	110 (45.6)	-	
Melanoma of skin	19 (7.9)	-	
Kidney and renal pelvis	13 (5.4)	-	
Urinary bladder	13 (5.4)	-	
Lung and bronchus	5 (2.1)	-	
Prior cancer stage			
Stage 0/I	141 (58.5)	-	
Stage II	31 (12.9)	-	
Stage III	22 (9.1)	-	
Stage IV	25 (10.4)	-	
Missing	22 (9.1)	-	

^a^ T-test p-value, ^b^ Fisher's test p-value, all other statistical tests were Chi-square test #: These men may have an unknown primary with only lymph nodes involved. Their diagnosis was confirmed by positive pathology Note: Missing values were not included in Chi-square or Fisher's analysis

**Table 2:** Cumulative incidence of all-cause and breast cancer-specific death among men diagnosed with breast cancer.

Time (in years) since breast cancer diagnosis	All cause		Breast cancer-specific	
	With Prior cancer	Without Prior Cancer	With Prior cancer	Without Prior Cancer
	Cumulative incidence in percentage (95% confidence interval)			
1	8.7 (5.6, 12.7)	5.8 (4.7, 7.0)	2.9 (1.3, 5.6)	2.7 (2.0, 3.5)
2	15.4 (11.2, 20.3)	11.0 (9.6, 12.6)	4.2 (2.1, 7.2)	5.4 (4.4, 6.6)
3	26.7 (21.3, 32.4)	16.2 (14.5, 18.0)	8.3 (5.3, 12.3)	8.0 (6.7, 9.3)
4	33.4 (27.5, 39.5)	22.0 (20.1, 24.1)	10 (6.6, 14.2)	10.9 (9.4, 12.4)
5	41 (34.7, 47.2)	27.2 (25.1, 29.4)	11.7 (8, 16.2)	13.5 (11.9, 15.2)

**Table 3:** Hazard ratios and subdistribution hazard ratios demonstrating association of prior cancer and survival.

	No. of breast cancer-specific deaths	Breast cancer- specific survival	No. of all-cause deaths	Overall survival	No. of breast-cancer specific deaths	Breast cancer- specific survival	No. of all-cause deaths	Overall survival
	Crude HR (95% CI)				^$^Adjusted HR (95% CI)			
**Full sample of male breast cancer patients demonstrating association of prior cancer compared to those with no prior cancer (n=1923)**								
Prior cancer	38	0.80 (0.57, 1.12)	150	1.7 (1.39, 1.97)*	34	0.94 (0.66, 1.35)	139	1.31 (1.09, 1.57)
No prior cancer	327	Ref.	754	Ref.	295	Ref.	701	Ref.
**Subgroup analyses of male breast cancer patients demonstrating association of prior prostate cancer compared to those without prior cancer (n=1792)**								
Prior prostate cancer	12	0.55 (0.31, 0.99)	59	1.32 (1.02, 1.70)	10	0.57 (0.31, 1.05)	55	1.01 (0.77, 1.32)
No prior cancer	327	Ref.	754	Ref.	295	Ref.	701	Ref.
**Subgroup analyses demonstrating association of time elapsed between prior cancer and index breast cancer with survival (n=241)**								
≤5 years	22	1.07 (0.57, 2.03)	85	1.01 (0.74, 1.39)	18	0.44 (0.20, 0.94)	77	1.08 (0.77, 1.52)
>5 years	16	Ref.	65	Ref.	16	Ref.	62	Ref.

HR: The HR for breast cancer specific survival represents subdistribution hazard ratio CI: Confidence Interval $ Adjusted model for age, breast cancer stage, and tumor size. *Indicates p-value <0.01

## Data Availability

The datasets were derived from sources in the public domain: [Surveillance, Epidemiology, and End Results (SEER), Surveillance, Epidemiology, and End Results Program (cancer.gov)].
